# Effect of a Probiotic Mixture in Captive Cheetahs (*Acinonyx Jubatus*) with Gastrointestinal Symptoms—A Pilot Study †

**DOI:** 10.3390/ani12030395

**Published:** 2022-02-07

**Authors:** Sara Mangiaterra, Anne Schmidt-Küntzel, Laurie Marker, Alessandro Di Cerbo, Renato Piccinini, Davide Guadagnini, Maria Elena Turba, Sara Berardi, Livio Galosi, Silvia Preziuso, Matteo Cerquetella, Giacomo Rossi

**Affiliations:** 1 School of Biosciences and Veterinary Medicine, University of Camerino, 62024 Matelica, MC, Italy; alessandro.dicerbo@unicam.it (A.D.C.); livio.galosi@unicam.it (L.G.); silvia.preziuso@unicam.it (S.P.); matteo.cerquetella@unicam.it (M.C.); giacomo.rossi@unicam.it (G.R.); 2 Cheetah Conservation Fund, Otjiwarongo 9000, Namibia; genetics@cheetah.org (A.S.-K.); director@cheetah.org (L.M.); 3 Falconara Zoo Park, 60015 Falconara, AN, Italy; parcozoofalconara@libero.it; 4 Parco Faunistico Le Cornelle, 24030 Valbrembo, BG, Italy; davide.guadagnini@aulss9.veneto.it; 5 Genefast Laboratory, 47122 Forlì, FC, Italy; me.turba@genefast.com; 6 DVM Freelance Consultant, 62100 Macerata, MC, Italy; sberardi@ormendes.ch

**Keywords:** cheetah, gastroenteric disorders, *Helicobacter* spp., probiotics

## Abstract

**Simple Summary:**

In recent years, many studies have demonstrated the effectiveness of probiotics in acute and chronic gastrointestinal disorders in both humans and animals. The objective of this study is to evaluate the effect of a specific probiotic mixture in cheetahs. In the wild, cheetah populations have drastically reduced due to habitat destruction, human–wildlife conflict and illegal wildlife trade. In captivity, chronic gastrointestinal diseases have a high prevalence. Based on our results, it can be concluded that probiotics may be helpful as a dietary supplement in cheetahs suffering from gastrointestinal disease.

**Abstract:**

Cheetahs (*Acinonyx jubatus*) are classified as “vulnerable” species due to the low numbers persisting in the wild. Gastrointestinal diseases are very common in this species when they are kept in captivity, in particular gastritis. Clinical signs are predominantly characterized by vomiting, diarrhea, weight loss and anorexia. In this study, we evaluated the efficacy of a multi-strain probiotic in two groups of cheetahs: Group A (*n* = 4)—rescued cheetahs housed at the Cheetah Conservation Fund (Otjiwarongo, Namibia); Group B (*n* = 9)—captive cheetahs housed in Italian zoos. Animals showed gastrointestinal signs of different severity, and were positive for *Helicobacter* spp., detected by PCR in stool samples. Two sachets of probiotic formulation were administered to all cheetahs once a day for 21 consecutive days. Clinical conditions (appetite loss, vomiting, stool consistency and Body Condition Score) before (T0) and after 21 days of probiotic administration (T1) were then compared using a simplified Feline Chronic Enteropathy Activity Index (FCEAI) score. A slight but not significant improvement in the scores was observed in Group A, which had mild intestinal symptoms, while a significant decrease in vomiting and stool consistency (**p < 0.01) scores was observed in Group B, which had more pronounced symptoms. Results suggest that high concentrations of live probiotics can be of help in managing gastrointestinal signs in cheetahs.

## 1. Introduction

The cheetah is the only species of the genus *Acinonyx* and is classified as “vulnerable” due to the low number of individuals (~7100 mature individuals) persisting in the wild [1]. In this species, gastritis has been an important clinical disease reported predominantly in the captive population; in the 1990s it was noted in approximately 91% of the North American captive cheetahs studied [2]. Chronic gastritis may predispose cheetahs to other chronic pathologies up to the death of the animal and is often associated with the presence of gastric *Helicobacter* organisms [2,3]. The severity of the clinical presentation is different between captive and free-ranging animals [2,3]: vomiting, diarrhea, weight loss and anorexia are observed in cheetahs hosted in zoos, whereas few to no symptoms are evident in free-ranging animals. Two *Helicobacter* species have mainly been identified in cheetahs with gastritis: *H. acinonychis* (or *H. acinonyx*), associated with lymphoplasmacytic gastric infiltration, lymphoid follicles with hyperplastic gut-associated lymphoid tissue (GALT) and evidence of gastric reflux, erosion and necrosis, and *H. heilmannii*, which seems to be less frequently associated with gastric disease [4]. Conventional therapy for the treatment of gastritis in cheetahs, as in other animals, involves the use of antibiotic therapy [5,6], although it is not recommended for mild cases [7].

Today, in the “post-antibiotic era”, more attention is paid to new therapeutic approaches, such as the use of probiotics and prebiotics [8,9]. These nutraceutical products have multiple functions in gastrointestinal disorders such as acute diarrhea, diarrhea associated with antibiotic use, gastrointestinal functional disorders and chronic inflammatory diseases [10,11,12]. Both immunological and non-immunological inhibitory action of probiotics against *H. pylori* have been suggested to enhance the standard therapy in humans [13]: while administration of probiotics alone did not eradicate *H. pylori*, a reduction in levels was detected in several studies; the use of probiotics as adjuvant therapy to antibiotic treatment showed an increased eradication rate of *H. pylori* and a reduction in treatment-associated side effects [14,15]. In animal models, probiotics were suggested to increase the concentration of anti-*H. pylori* IgA and IgG and to modulate cytokine secretion and mRNA expression [16,17,18,19]. To have measurable effects, treatment length of a minimum of two consecutive weeks was recommended [20]. Researchers suggested that the genus *Lactobacillus* can participate in *Helicobacter* eradication [21,22], competing with *H. pylori* strains for adhesion sites [23] and producing metabolites or interleukins that may decrease the number or mucosal concentration of spiral bacteria [24,25,26,27,28]. Similarly, the ability of genera *Streptococcus* and *Bifidobacterium* to inhibit bacterial adhesion and inflammatory response in gastric mucosa has been demonstrated [29,30,31]. Studies also suggested that, during *Helicobacter* infection, *Bifidobacterium longum* and *Saccharomyces boulardii* can reduce the frequency of diarrhea and in some cases help eradicate *Helicobacter* spp. [32,33,34].

There are no data on the impact of probiotics on *Helicobacter* spp. or gastritis in cheetahs to date. However, in a study conducted in South Africa, administration of probiotics to a group of 27 young cheetahs qualified as healthy with episodic diarrhea showed an increase in body weight compared to the control group and a reduction in the emission of feces with mucus and blood during the period of treatment [35]. The aim of our study was to evaluate the efficacy of a probiotics blend on digestive health in cheetahs. Study animals included wild-born rescue cheetahs housed at the Cheetah Conservation Fund in Namibia and captive-born cheetahs housed at two Italian zoos. We detected an improvement in the observed digestive parameters with administration of probiotics.

## 2. Materials and Methods

### 2.1. Study Population

Two groups of cheetahs were considered for inclusion in the study. Group A included 9 (8 males, 1 female) rescued cheetahs living at the Cheetah Conservation Fund, Otjiwarongo, Namibia; Group B included 9 (6 males, 3 females) captive cheetahs hosted in 2 Italian zoos, Parco Zoo Falconara (AN) and Le Cornelle (BG). Animals in Group A were living in large enclosures in their natural environment and mainly fed with meat derived from ungulates (about 2 kg of ungulate meat on the bone with mineral supplements (™Predator Powder) for each animal) in a single meal six days out of seven. Cheetahs were chosen based on their behavior to ensure the possibility of administering probiotics and observing potential symptoms. These animals, aged between 5 and 9 years, were associated with a clinical history referring to rare episodes of vomiting and diarrhea (<1 episode per month) and did not all present symptoms at the time of the study. Animals in Group B were housed in on- or off-exhibit zoo enclosures and mainly fed with meat derived from rabbit or poultry (about 1.1 kg of meat for each animal with mineral supplements (™Predator Powder) for each animal) in a single meal per day. Of these, 3 cheetahs were over 10 years old, 4 between 5 and 10 years old and 2 under 3 years old. Seven out of the nine cheetahs had a clinical history associated with recurrent, variably frequent, severe episodes of vomiting, diarrhea (>1 episode per month) and weight loss (5–10% of weight loss). The other 2 cheetahs had a recent history of abnormal fecal consistency (slightly or very soft with mucus and increased frequency). Animals that underwent antibiotic therapy in the past 30 days were excluded.

### 2.2. Verification of Helicobacter spp. Presence

Fecal samples were collected three days before starting the treatment (T0). Individual fecal samples were collected for each animal within twelve hours of defecation and frozen at −20 °C until analysis. For animals housed in the same enclosure, markers were fed individually the day preceding sample collection to allow identification of the feces.

DNA was extracted from 200 mg ± 20 mg fecal samples using a QIAamp DNA Stool Mini Kit (Qiagen, Hilden, Germany) according to the manufacturer’s protocol. PCR analysis was carried out similarly to Camargo et al. [36], with the following modification: a touch-down protocol was used for annealing temperature (1 °C drop per cycle for 10 cycles, from 60 to 50 °C, followed by 30 cycles with 50 °C annealing temperature) as it yielded better results. Primers F, AACGATGAAGCTTCTAGCTTGCTA, and R, GTGCTTATTCGTGAGATACCGTCAT, were used to amplify a 399 bp sequence of the 16S rRNA gene of *Helicobacter* spp. [37]. Amplification of *Helicobacter* spp. was verified by gel electrophoresis on a 1.5% agarose gel. Ten microliters of the amplification products were visualized with ethidium bromide (0.5 mg/mL in the gel) or GelRed^®^ (combined with the sample at 1.5×).

One individual was subjected to digestive endoscopy and showed evidence of chronic gastro-esophagitis; for this individual, a gastric biopsy of the gastric mucosa was additionally assessed with histopathology.

### 2.3. Clinical Evaluation

Clinical evaluations of all PCR-positive individuals in both groups were performed by the same observer, once before (T0) and once after the treatment (T1). To evaluate clinical condition, a clinical score was assigned to each patient using a simplified Feline Chronic Enteropathy Activity Index (FCEAI) scoring system [38]. The “simplified” FCEAI score was limited to the published gastrointestinal parameters, which can be obtained non-invasively: appetite, vomiting, stool consistency and weight loss (Table 1). Attitude was not included as it is more difficult to evaluate in non-domestic species, and the observer was not sufficiently familiar with the animals to assess it. Weight loss had to be substituted with Body Condition Score (BCS) as weights were not available at the time of the study. The two populations were then evaluated, comparing individual parameters and simplified FCEAI scores before and after probiotics administration.

### 2.4. Probiotics Administration

A specific formulation of probiotics, the SLAB51^®^, consisting of 8 different strains of live bacteria (*S. thermophilus* DSM32245/CNCM I-5570, *L. acidophilus* DSM32241/CNCM I-5567, *L. plantarum* DSM32244 CNCM I-5569, *L. paracasei* DSM32243/CNCM I-5568, *L. helveticus* DSM32242/CNCM I-5573, *L. brevis* DSM27961/CNCM I-5566, *B. lactis* DSM32246/CNCM I-5571, *B. lactis* DSM32247/CNCM I-5572), was administered to all PCR-positive cheetahs in the study. Two probiotic sachets containing at least 200 billion bacteria were administered once a day for 21 consecutive days (T0 to T1).

### 2.5. Statistical Analysis

Data were analyzed using GraphPad Prism 8 software (GraphPad Software, Inc., La Jolla, CA, USA) and reported as the mean ± standard error of the mean. Differences in the parameters making up the simplified FCEAI score (appetite, vomiting, stool consistency and BCS) and the simplified FCEAI score itself within the same group at T0 vs. T1 (following probiotic administration) were analyzed using a Wilcoxon matched-pair signed-rank test. A *p < 0.05 was considered significant.

## 3. Results

Out of 18 cheetahs selected for the study, 13 were PCR-positive for fecal *Helicobacter* spp. presence. The PCR-positive individuals included four of the nine individuals in Group A and all of the nine individuals in Group B. Helicobacter presence was additionally confirmed with histopathology in the gastric mucosa of an individual of Group B subjected to digestive endoscopy. All 13 PCR-positive cheetahs were included in the probiotic trial.

Within each individual, the score of every assessed gastrointestinal parameter either improved or remained constant when comparing values from after the trial (T1) to values from before the trial (T0). Scores of individual parameters from Group A ranged from 0 to 2 (out of 3) pre-treatment and 0 to 1 post-treatment, with the overall mean composite simplified FCEAI score reducing from 2.00 to 0.50 (out of 12) between T0 and T1 (Table 2). Scores of individual parameters from Group B ranged from 0 to 3 (out of 3) pre-treatment and 0 to 2 post-treatment, with the overall mean composite simplified FCEAI score reducing from 5.44 to 1.56 (out of 12) between T0 and T1 (Table 3).

**Figure 1:**
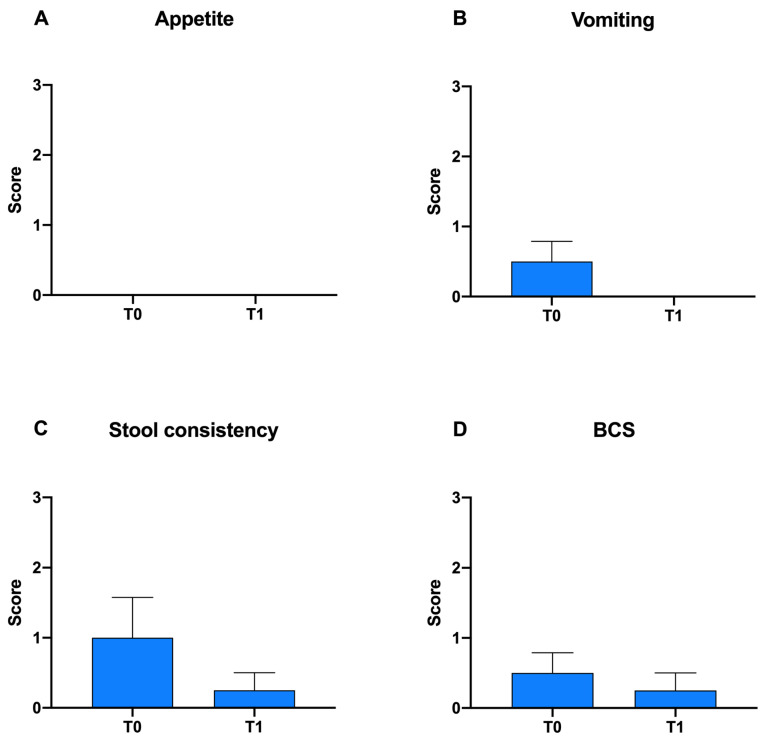
Graphical representation of the gastrointestinal parameters appetite (**A**), vomiting (**B**), stool consistency (**C**) and BCS (**D**), which make up the simplified FCEAI score within Group A at T0 vs. T1 following probiotic administration.

In Figure 1, differences in appetite, vomiting, stool consistency and BCS score within Group A at T0 vs. T1 are reported. A slight but not significant decrease was observed in vomiting, stool consistency and BCS score after probiotic administration (Figure 1B–D).

**Figure 2:**
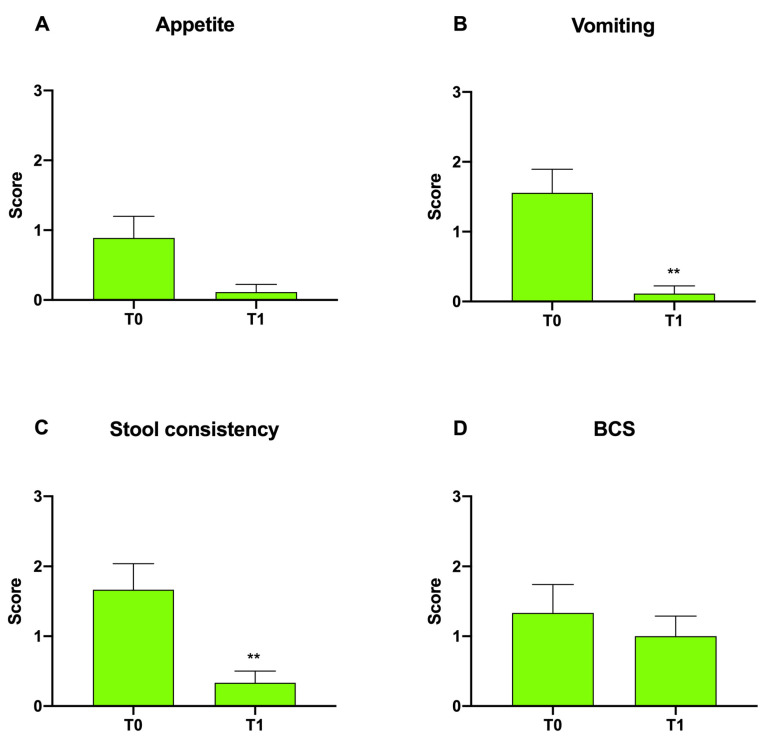
Graphical representation of the appetite (**A**), vomiting (**B**), stool consistency (**C**) and BCS (**D**) modified FCEAI score within both Group B at T0 vs. T1 following probiotic administration (**p < 0.01).

For Group B, a measurable but insignificant decrease was found in appetite and BCS scores (Figure 2A,D). On the other hand, a significant decrease in vomiting and stool consistency scores was observed in Group B at T1 with respect to T0, from 1.56 ± 0.33 to 0.11 ± 0.11 (**p < 0.01) and from 1.67 ± 0.37 to 0.33 ± 0.17 (**p < 0.01), respectively (Figure 2B,C).

**Figure 3:**
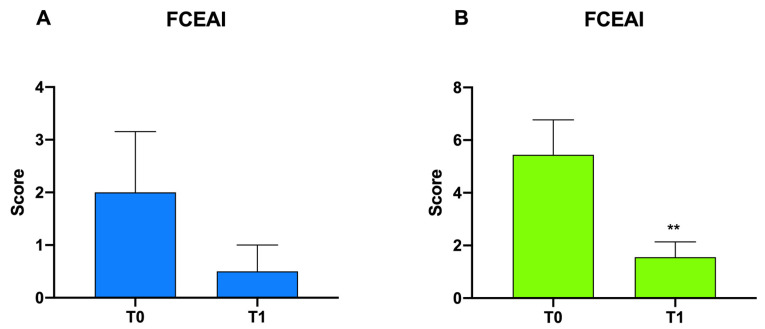
Graphical representation of the FCEAI score in Group A (**A**) and Group B (**B)** following probiotic administration; **p < 0.01.

In Figure 3, differences in FCEAI scores in Group A and B at T0 vs. T1 are reported. A measurable but insignificant decrease in scores was observed in Group A after probiotic administration, while a significant reduction in scores in Group B, from 5.44 ± 1.32 to 1.56 ± 0.58 (**p < 0.01), was observed.

## 4. Discussion

In this study, we evaluated the efficacy of a probiotics blend on digestive health in cheetahs in two cheetah populations. We compared the clinical conditions pre- and post- treatment using a simplified FCEAI score and detected an improvement in the clinical score with administration of probiotics [38]. For this study, the decision was made not to subject study animals to invasive procedures involving anesthesia or blood draws. As such, gastric biopsies were not performed, and therefore, gastritis diagnosis was not established; however, clinical evaluation of symptoms provided evidence of gastrointestinal distress, which was quantified using a simplified FCEAI score. The simplified FCEAI score accounts for a maximum of 12 out of the 20 available points in the full FCEAI score. In 95% of cases, chronic gastritis and esophagitis in cheetah are associated with the presence of gastric *Helicobacter* organisms, which usually determines different clinical outcomes depending on whether the animal lives in captivity or in the wild. However, gastritis is a multifactorial pathological process, and different factors contribute to the disease [39]. Definitive diagnosis of gastritis is made by biopsy and histological examination; however, these are not always feasible, so in veterinary medicine, treatment is often based on clinical symptoms alone. Symptoms observed in the course of gastritis in cheetahs are characterized by sialorrhea, vomiting, decreased appetite and, in chronic conditions, weight loss [40]. An improvement in symptoms is usually considered as indicative of likely treatment success. In the present study, cheetahs responded positively to probiotics administered for 21 days, and a statistically significant reduction in simplified FCEAI scores was observed in zoo cheetahs (Group B), which started off with more severe symptoms.

Statistical significance was not achieved to support the clinical improvement observed in study Group A. This is likely in part attributable to the low simplified mean FCEAI score of Group A at T0 (mean of 2.0 out of 12), which included two individuals with a simplified FCEAI score of 0. Indeed, only a minor measurable improvement can be expected when symptoms are relatively benign at the beginning of the study. Despite the lack of statistical support, all values remained either the same or improved after the treatment (Table 2), suggesting that a larger sample size may have been able to provide statistical support for the improvement observed. For Group B, both vomiting and stool consistency, as well as the combined FCEAI score, showed statistically significant improvement. The less marked improvement of the BCS is not surprising, as assimilation of nutrients is expected to take longer before impacting overall BCS than improvement in variables more directly linked to digestive health such as appetite, vomiting and stool consistency. In our current study, the mean score attributed to BCS improved from 1.33 to 1.00. We suggest that a larger sample size or longer time period could have yielded sufficient data to provide statistical support for the improvement in BCS, as well. Thus, the results of this pilot study are very encouraging.

In particular, in suspected gastritis cases that are not confirmed by gastric biopsies, treatment choices are a challenge, as classic antibiotic-based gastritis treatment can lose efficacy over time and can induce side effects of its own. Since 1996, international guidelines in human medicine for *Helicobacter* spp. infection established a conventional therapy involving the use of a proton pump inhibitor associated with amoxicillin and clarithromycin [41]. In cheetahs, treatment for gastritis caused by *Helicobacter* spp. includes the use of lansoprazole, clarithromycin and amoxicillin or omeprazole; clarithromycin and amoxicillin; or tetracycline, metronidazole and Bismuth subsalicylate [42]. Unfortunately, in the long term, the treatment can be ineffective [43], likely due to the increase in resistance to antibiotics similar to what happens in other animals and humans. In addition, these treatments can induce gastrointestinal side effects themselves [43]. For this reason, over the years, there has been a growing interest in new approaches to substitute, supplement or counteract side effects of antibiotic treatments, such as complementary feeds or probiotics. Bacterial strains in probiotics are selected by biochemical and genetic characteristics as well as safety for the health of the host [20]. Some probiotic strains have been associated with inhibitory functions against *H. pylori*, including *Saccharomyces boulardii*, *Lactobacillus* and *Bifidobacterium* [23,44,45,46]. Such probiotics have been ascribed the ability to modulate the infection through immunological and non-immunological mechanisms, helping eradicate or reduce collateral effects caused by antibiotic therapy [16]. The use of probiotics would thus be beneficial if their effect can be demonstrated through clinical studies.

The conclusions of the current study are limited by the small sample size, which is largely attributable to the species involved and the challenge of enrolling large numbers of cheetahs; however, despite this limitation, clinical improvements were observed, of which several were supported by statistical power, suggesting that the improvements were meaningful and that our results represent an interesting starting point that warrants further investigation. It is important to note that no adverse clinical effects were observed in either study group, indicating that there is no current contraindication to proceeding with additional research on the topic, as the benefits appear to outweigh any risk based on clinical signs. Future studies should include negative controls with similar symptomatology as the cases not yet subjected to probiotics treatment, to ensure that improvements could not be caused by alternative factors. In addition, if possible, additional parameters of the FCEAI score should be included to allow for a more thorough characterization of digestive health. Attitude/activity could be assessed if the observer is more familiar with the animal; endoscopy and blood collection would allow the inclusion of total protein, alanine transaminase, serum alkaline phosphatase and phosphorous. However, it is noteworthy that the parameters selected in this study already represent 12 out the 20 points of a full FCEAI score. Including attitude/activity would increase the maximum attainable score to 15 without the need for invasive sample collection; therefore, the risks and benefits of including invasive measures to obtain additional data points need to be carefully weighed.

## 5. Conclusions

This study was conducted to evaluate the efficacy of a specific probiotic mixture in improving clinical gastrointestinal signs in cheetahs. Our results were encouraging, especially in a zoo setting (Group B), with a mean pre-treatment simplified FCEAI score of 5.4 (out of 12). A significant decrease in individual parameter scores for vomiting and stool consistency and in the simplified FCEAI score were observed in that Group at T1 with respect to T0, reflecting an improvement in clinical condition. The present pilot study suggests that probiotics administration can modulate the gastrointestinal environment, inducing an improvement of symptoms in diseased subjects. Further studies are needed to confirm present results and to evaluate whether probiotics may be beneficial as additive or alternative therapy during *Helicobacter* infection in cheetahs.

## Figures and Tables

**Table 1:** Simplified FCEAI score used for clinical evaluation modified from [38].

Score	Appetite	Vomiting	Stool Consistency	Weight Loss
0	Normal	None	Normal: well-formed feces	None
1	Slight decrease	Mild (once a week)	Normal: well-formed feces	Mild (<5%)
2	Moderate decrease	Moderate (twice a week)	Very soft, moderately increased frequency	Moderate (5–10%)
3	Severe decrease	Severe (>2–3 times a week)	Watery diarrhea	Severe (>10%)

**Table 2:** Simplified FCEAI score in Group A at T0 and T1.

Cheetah	Appetite		Vomiting		Stool Consistency		BCS		Simplified FCEAI Score	
	T0	T1	T0	T1	T0	T1	T0	T1	T0	T1
1	0	0	1	0	2	0	1	0	4	0
2	0	0	1	0	2	1	1	1	4	2
3	0	0	0	0	0	0	0	0	0	0
4	0	0	0	0	0	0	0	0	0	0
Mean values	0.00	0.00	0.50	0.00	1.00	0.25	0.50	0.25	2.00	0.50

**Table 3:** Simplified FCEAI score in Group B at T0 and T1.

Cheetah	Appetite		Vomiting		Stool Consistency		BCS		Simplified FCEAI Score	
	T0	T1	T0	T1	T0	T1	T0	T1	T0	T1
1	2	0	3	0	3	1	3	2	11	3
2	2	0	3	0	3	1	3	2	11	3
3	0	0	2	0	2	0	2	1	6	1
4	0	0	1	0	1	0	0	0	2	0
5	0	0	1	0	0	0	0	0	1	0
6	0	0	1	0	1	0	0	0	2	0
7	1	0	1	0	1	0	1	1	4	1
8	1	0	0	0	1	0	1	1	3	1
9	2	1	2	1	3	1	2	2	9	5
Mean values	0.89	0.11	1.56	0.11	1.67	0.33	1.33	1.00	5.44	1.56

## Data Availability

Data are contained within the article. Some data included in the present manuscript have already been presented as abstract at XII Congress YABOUMBA WORLD. Exotic, Zoo and Wild Animals Conservation, Medicine and Surgery, Paris, 20–21 February 2020 [“Administration of the probiotic slab51 in captive and rescued cheetahs (*Acinonyx jubatus*) with gastrointestinal disorders: a clinical evaluation”, Mangiaterra, 2020].
